# Supplementary material for: A succession of two viral lattices drives vaccinia virus assembly
Source: PLoS Biol. 2023 Mar 2;21(3):e3002005. doi: 10.1371/journal.pbio.3002005 (PMC10013923; doi:10.1371/journal.pbio.3002005)
Supplement: S1 Table — Sample thickness was measured manually in the regions corresponding to the main figure tomograms using IMOD. In many cases, the presence of viral particles locally increases the thickness of the cell. For the larger regions in Fig 1B and 1C, a thickness range is provided in the corresponding figure legend (see Fig 1B and 1C). (DOCX) [file pbio.3002005.s001.docx]

**Supplementary Table 1. Sample thickness of main figure tomograms.**

| Figure | Sample thickness at the region shown (nm) |
| --- | --- |
| 2A | 282 |
| 2B | 340 |
| 2C (left) | 348 |
| 2C (right) | 299 |
| 2D | 326 |
| 2E | 227 |
| 3A | 332 |
| 4A | 295 |
| 4C (top) | 328 |
| 4C (bottom) | 369 |
| 5A | 287 |
| 5B | 369 |
| 6 | 195 |
| 7A (left) | 287 |
| 7A (middle) | 309 |
| 7A (right) | 323 |
| 9 (far left) | 364 |
| 9 (left) | 287 |
| 9 (right) | 338 |
| 9 (far right) | 301 |
