# Supplementary material for: A succession of two viral lattices drives vaccinia virus assembly
Source: PLoS Biol. 2023 Mar 2;21(3):e3002005. doi: 10.1371/journal.pbio.3002005 (PMC10013923; doi:10.1371/journal.pbio.3002005)
Supplement: S2 Table — (A) The middle-view perimeters, IV diameter, the major axis of IMV and EEV/CEV are shown, together with the standard error of the mean and the number of viral particles measured. The intermediate and minor axes of IMV are also shown, as well as the IEV axes. Finally, the calculated volume of IV, IMV, and IEV are provided. See Methods for details of dimension calculations. (B) For the thickness of the inner wall and the outer layer, 5 different IMV were measured by tracing 5 different lines for each IMV, which correspond to the 5 measurements shown. To estimate the distance between the palisade and the viral membrane (with no lateral bodies), 5 IMV were used. For the distance between the palisade and the plasma membrane through lateral bodies 1 IMV and 4 EEV were included in the quantification. All values correspond to nm. (DOCX) [file pbio.3002005.s002.docx]

**Supplementary Table 2. Measurements and calculations of virion dimensions.**

**A.**

| Middle view perimeter | Average (nm) | SEM |  |
| --- | --- | --- | --- |
| IV | 1106.39 | 8.58 | n = 15 |
| IMV | 1087.75 | 11.25 | n = 19 |
| EEV / CEV | 1074.46 | 7.09 | n = 20 |

| Diameter / major axis | Average (nm) | Standard Error of the Mean (SEM) |  |
| --- | --- | --- | --- |
| IV | 351.89 | 2.88 | n = 26 |
| IMV | 351.70 | 3.31 | n = 22 |
| EEV / CEV | 348.91 | 1.87 | n = 22 |

| IMV | Average (nm) | SEM |  |
| --- | --- | --- | --- |
| Intermediate axis | 280.53 | 3.66 | n = 19 |
| Minor axis* | 198.04 | 4.94 | n = 10 (3 IMV, 7 EEV) |

| IEV | Average (nm) | SEM |  |
| --- | --- | --- | --- |
| Major axis | 437.86 | 13.88 | n = 7 |
| Intermediate axis | 383.45112 | 10.95 | n = 7 |
| Minor axis (estimation) | 259.11 |  |  |

|  | Calculated volume (nm^3^) |
| --- | --- |
| IV | 2.28 x 10^7^ |
| IMV | 1.02 x 10^7^ |
| IEV | 2.28 x 10^7^ |

**B.**

| Inner wall thickness (nm) | IMV1 | IMV2 | IMV3 | IMV4 | IMV5 | Final average | Standard Deviation | SEM |
| --- | --- | --- | --- | --- | --- | --- | --- | --- |
| 1 | 4.89 | 4.12 | 2.08 | 4.43 | 3.72 |  |  |  |
| 2 | 4.29 | 3.88 | 4.28 | 3.20 | 2.71 |  |  |  |
| 3 | 3.54 | 4.21 | 4.75 | 3.11 | 4.78 |  |  |  |
| 4 | 3.25 | 3.48 | 3.61 | 2.38 | 4.28 |  |  |  |
| 5 | 3.91 | 4.02 | 2.81 | 6.05 | 4.20 |  |  |  |
| average | 3.98 | 3.94 | 3.51 | 3.84 | 3.94 | 3.84 | 0.19 | 0.09 |

| Outer layer thickness (nm) | IMV1 | IMV2 | IMV3 | IMV4 | IMV5 | Final average | Standard Deviation | SEM |
| --- | --- | --- | --- | --- | --- | --- | --- | --- |
| 1 | 7.24 | 8.15 | 6.14 | 6.90 | 5.61 |  |  |  |
| 2 | 7.51 | 7.14 | 7.25 | 6.52 | 7.31 |  |  |  |
| 3 | 7.48 | 6.55 | 5.86 | 6.37 | 5.05 |  |  |  |
| 4 | 6.40 | 7.12 | 6.03 | 6.96 | 6.63 |  |  |  |
| 5 | 5.58 | 5.77 | 7.71 | 6.28 | 7.11 |  |  |  |
| average | 6.84 | 6.95 | 6.60 | 6.61 | 6.34 | 6.67 | 0.11 | 0.24 |

| Palisade-to-viral membrane distance (nm) | IMV1 | IMV3 | IMV4 | IMV5 | Final average | Standard Deviation | SEM |
| --- | --- | --- | --- | --- | --- | --- | --- |
| 1 | 9.54 | 10.12 | 8.89 | 9.47 |  |  |  |
| 2 | 9.57 | 9.37 | 7.87 | 8.08 |  |  |  |
| 3 | 11.18 | 9.51 | 8.32 | 8.31 |  |  |  |
| 4 | 8.89 | 9.57 | 11.28 | 10.98 |  |  |  |
| 5 | 8.66 | 8.53 | 7.36 | 10.04 |  |  |  |
| average | 9.57 | 9.42 | 8.74 | 9.38 | 9.28 | 0.18 | 0.36 |

| Palisade to viral membrane through lateral bodies (nm) | IMV1 | EEV1 | EEV2 | EEV3 | EEV4 | Average | Standard  Deviation | SEM |
| --- | --- | --- | --- | --- | --- | --- | --- | --- |
| 1 | 53.20 | 50.35 | 43.45 | 52.01 | 41.76 |  |  |  |
| 2 | 31.93 | 48.19 | 42.39 | 62.64 | 39.35 | 46.53 | 8.60 | 2.72 |
