# Supplementary material for: A succession of two viral lattices drives vaccinia virus assembly
Source: PLoS Biol. 2023 Mar 2;21(3):e3002005. doi: 10.1371/journal.pbio.3002005 (PMC10013923; doi:10.1371/journal.pbio.3002005)
Supplement: S4 Table — (DOCX) [file pbio.3002005.s004.docx]

**Supplementary Table 4. Cryo-ET data collection and STA processing.**

|  | Palisade | | | | D13 |
| --- | --- | --- | --- | --- | --- |
|  | Combined | EEV/CEV | IMV | IEV |  |
| **Data collection** |  |  |  |  |  |
| Voltage (kV) | 300 | | | | |
| Cumulative electron dose (e / Å²) | 66.3 | | | | |
| Applied defocus (µm) | -8 | | | | |
| Pixel size (Å) | 4.31 | | | | |
| Tilt range (°) | ±57°, dose symmetric | | | | |
| **STA processing** |  |  |  |  |  |
| Map symmetry | C3 | C3 | C3 | C3 | C6 |
| Initial particle number | 182938 | 209703 | 80698 | 8256 | 341376 |
| Final particle number | 179822 | 149662 | 27590 | 5686 | 34092 |
| Map resolution (Å) (FSC=0.143) | 21.2 | 21.2 | 25.1 | 30.6 | 19.2 |
