# Supplementary figures and images for: A succession of two viral lattices drives vaccinia virus assembly
Source: PLoS Biol. 2023 Mar 2;21(3):e3002005. doi: 10.1371/journal.pbio.3002005 (PMC10013923; doi:10.1371/journal.pbio.3002005)

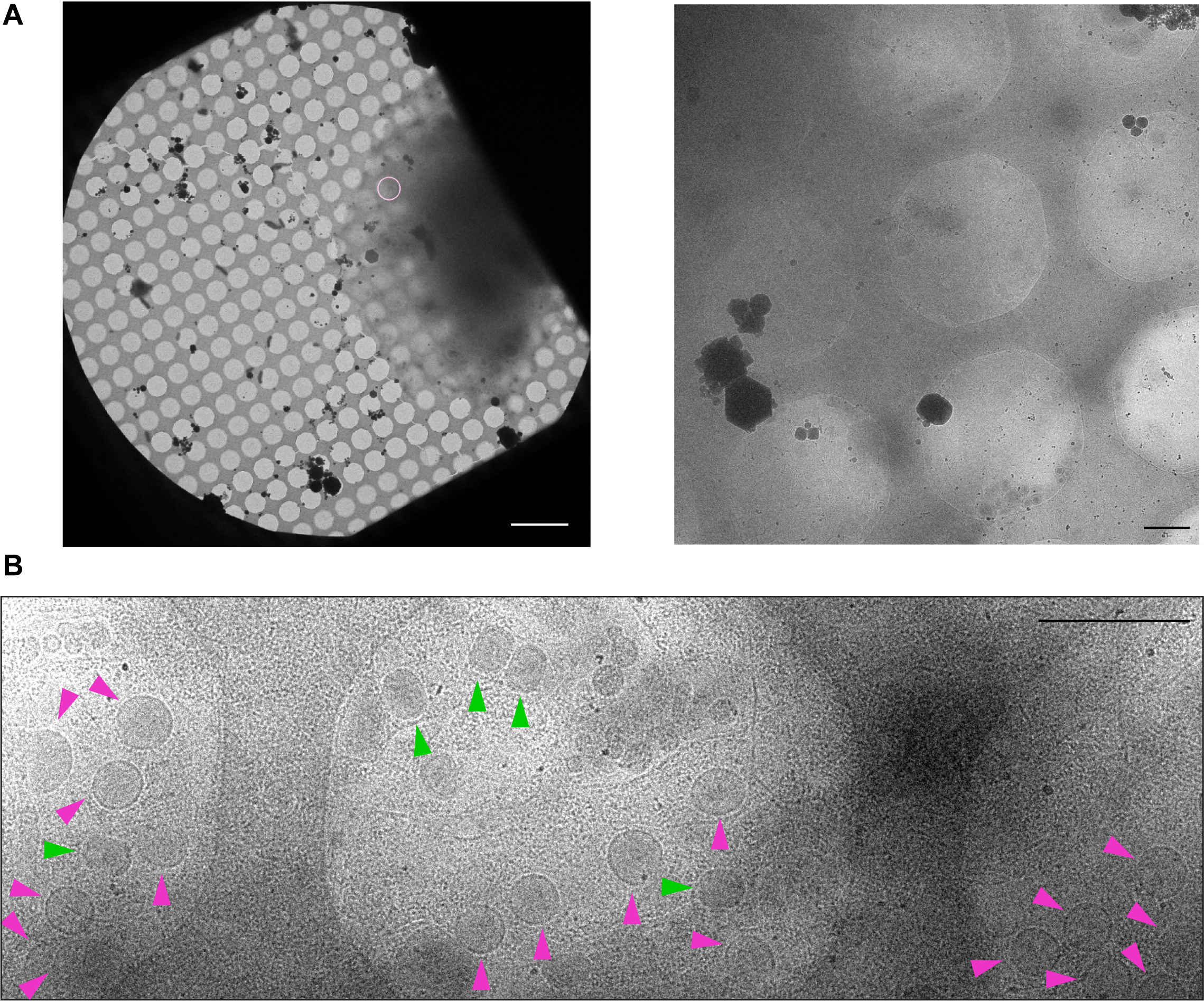

Supplement: S1 Fig — (A) The pink circle marks the position where the IV in Fig 1B were imaged. The image on the right is a magnification of the circled area and its surroundings. (B) A cryo-EM image showing IV (magenta arrow) and IMV (green arrow) visualised at low magnification in cells over holes in the carbon support film. Scale bars = 10 (left) and 1 μm (right) (A) and 1 μm (B). (TIF) [file pbio.3002005.s005.tif]

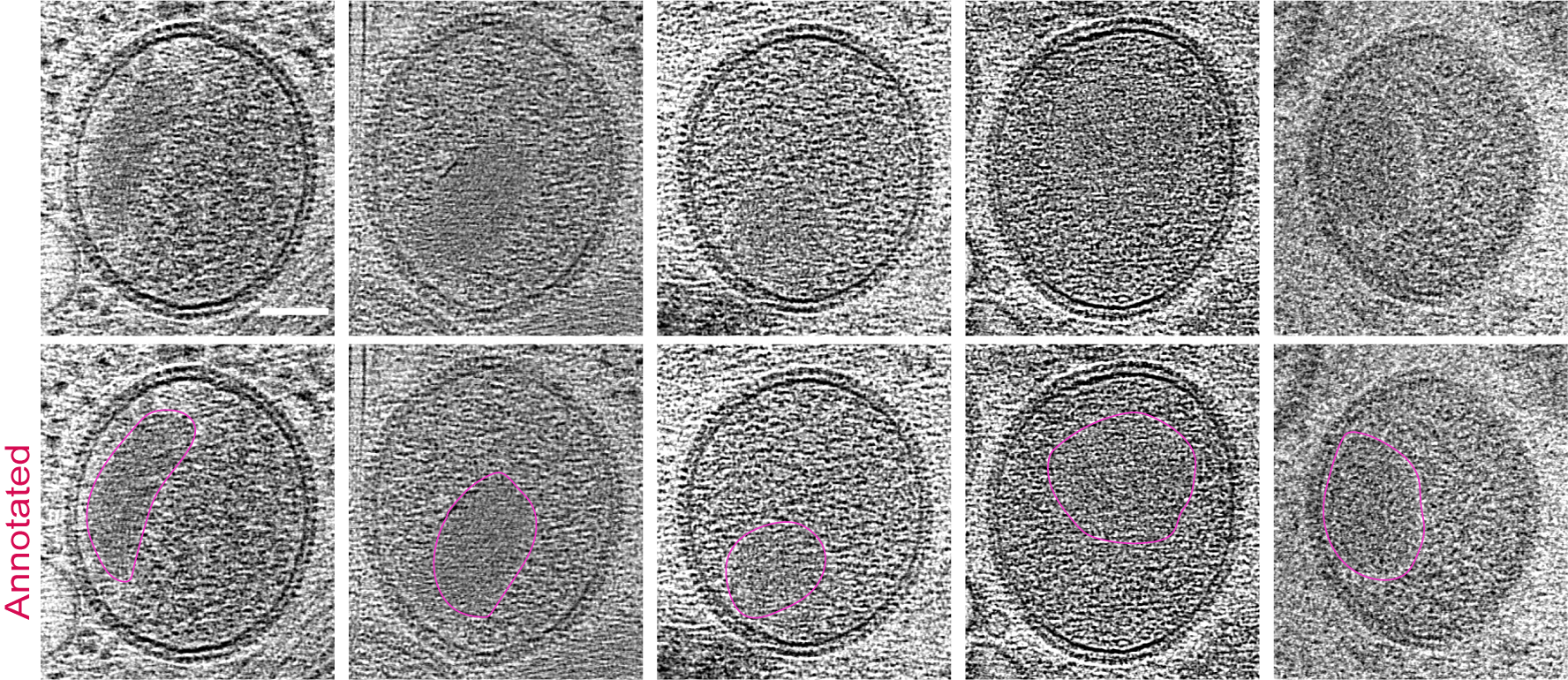

Supplement: S2 Fig — Tomographic sections showing IV with an internal condensed nucleoid outlined in magenta in the bottom row. Scale bar = 100 nm. (TIF) [file pbio.3002005.s006.tif]

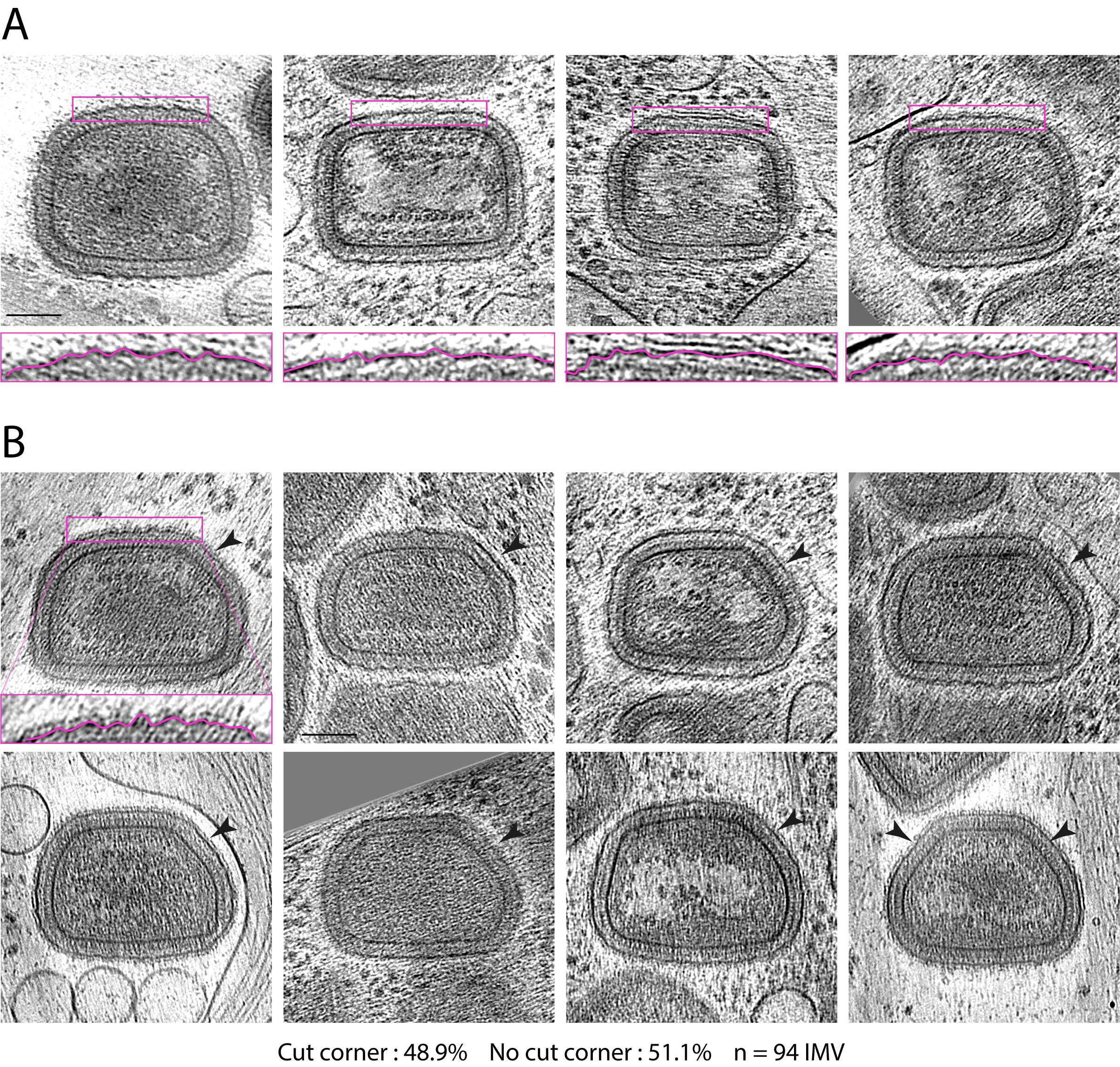

Supplement: S3 Fig — (A) Gallery of tomographic sections of IMV with no cut corner, with magnified regions (2×) to highlight the wrinkled viral membrane. (B) IMV with cut or flattened corner/s (black arrowheads). The magnified region of the first IMV shows the corrugation of the viral membrane, as in (A). Scale bars = 100 nm. (TIF) [file pbio.3002005.s007.tif]

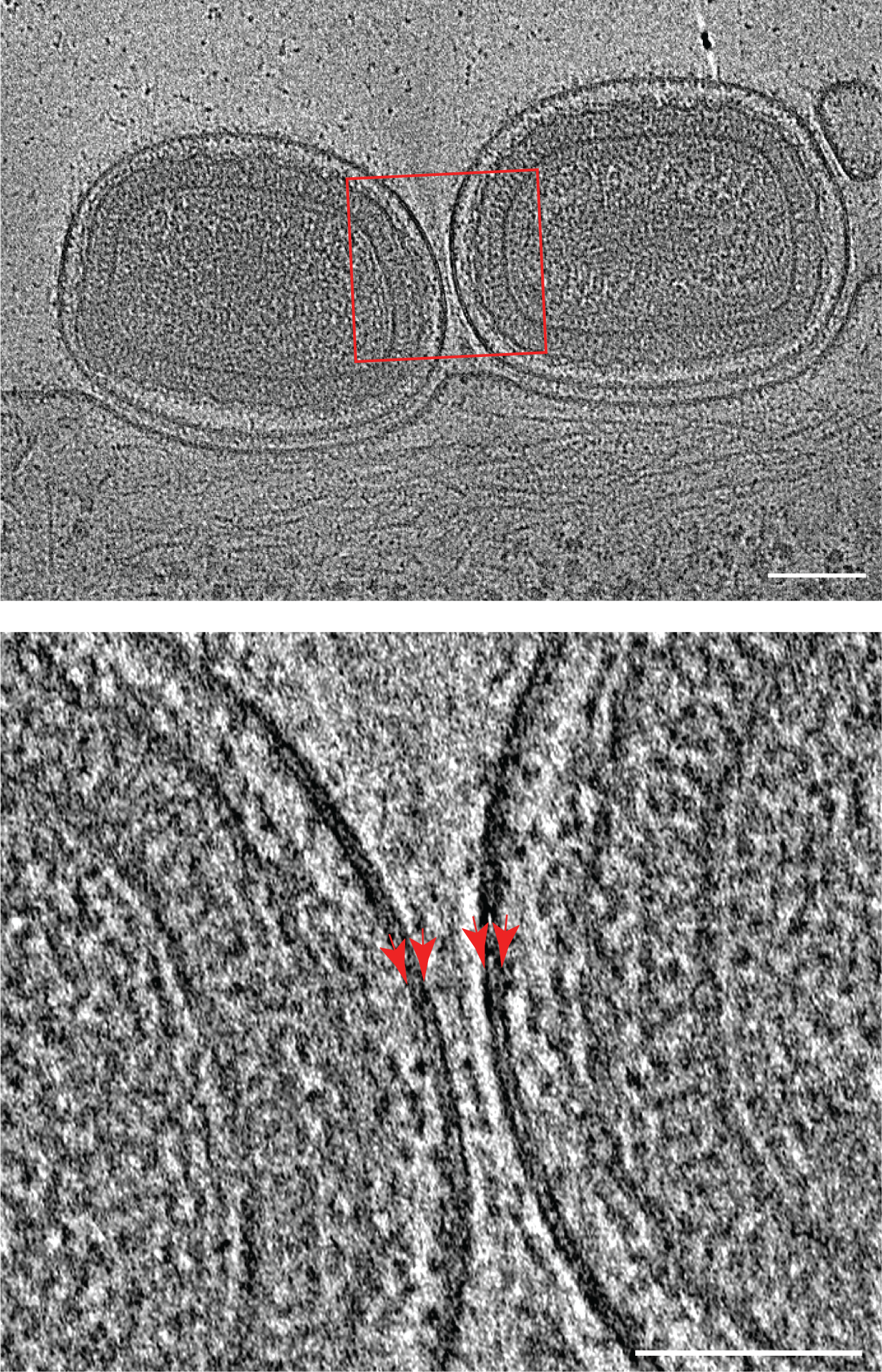

Supplement: S4 Fig — The CEV outer membrane in the unbinned tomogram appears as 2 parallel densities, consistent with the bilayer organisation of membranes. The CEV on the right corresponds to the one shown on Fig 7. The red square corresponds to the enlarged image in which arrowheads point to the 2 leaflets on the CEV outer membranes. Top scale bar = 100 nm. Bottom scale bar = 50 nm. (TIF) [file pbio.3002005.s008.tif]

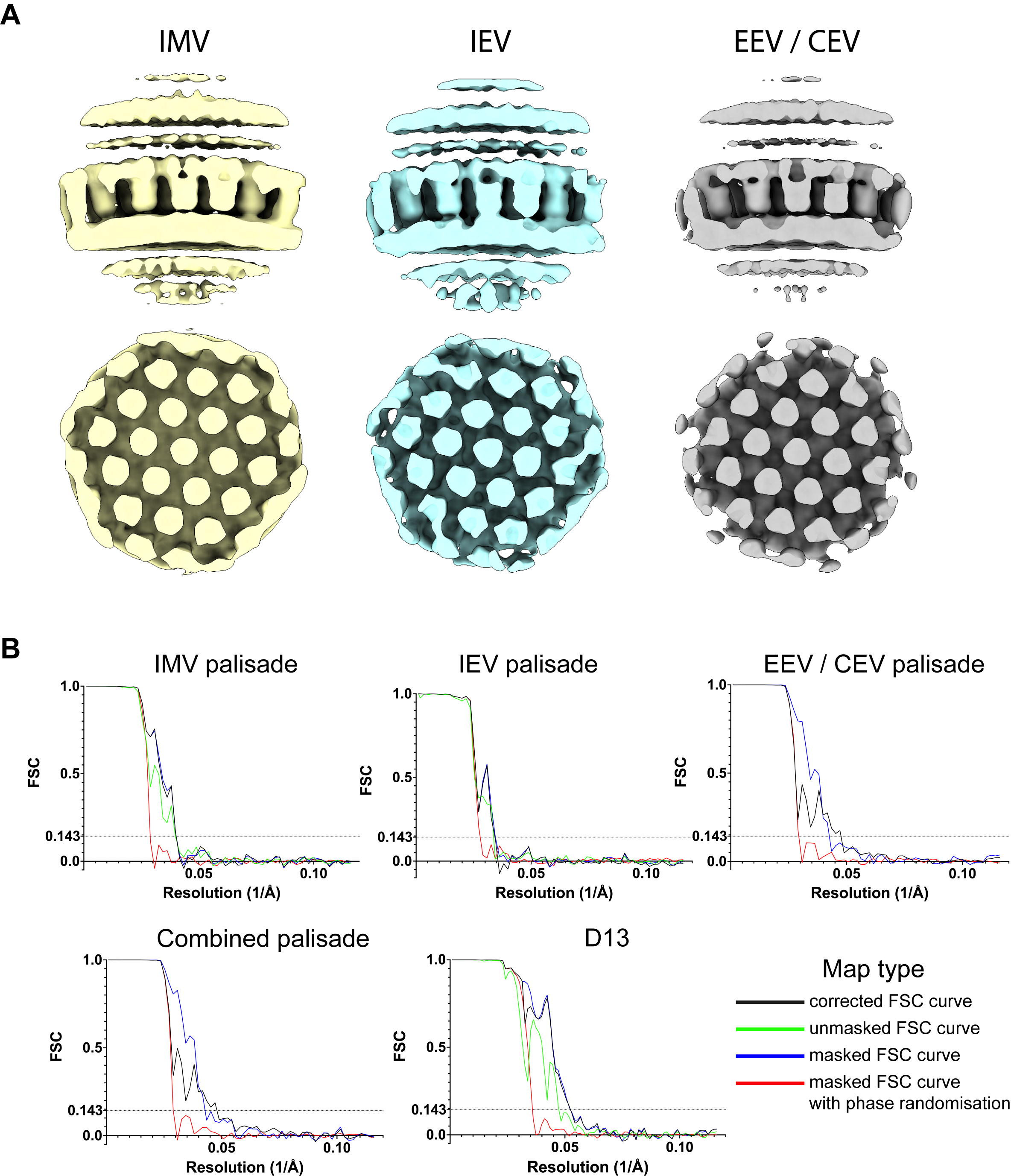

Supplement: S5 Fig — (A) Maps derived from subtomogram averaging IMV, IEV, or EEV/CEV. A surface (top) and a cut-section view (bottom) are shown. (B) Fourier shell correlation plots for palisade maps as well as for the D13 map. Curves are given for the corrected, unmasked, masked, and masked with phase randomisations calculations. (TIF) [file pbio.3002005.s009.tif]

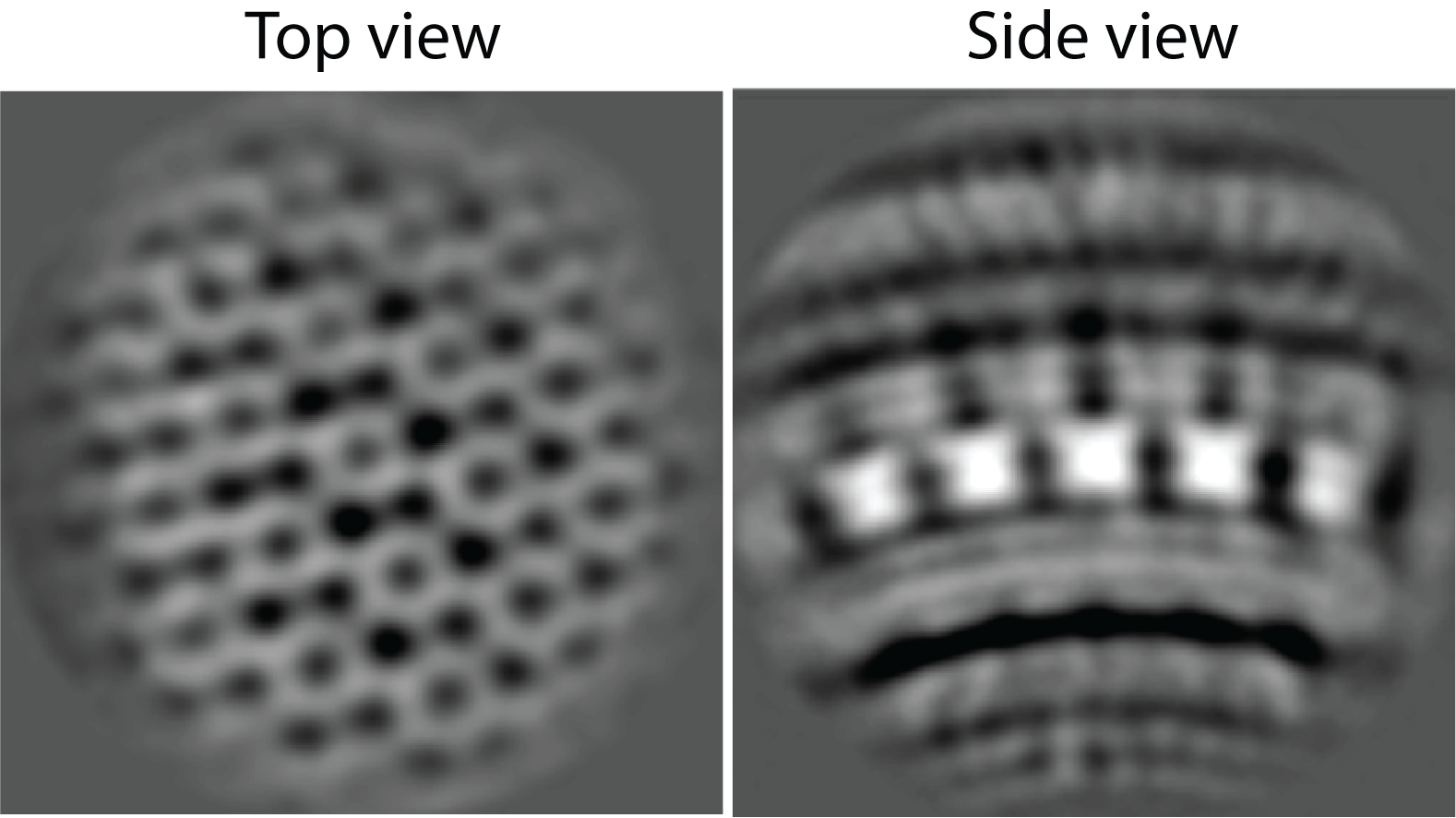

Supplement: S6 Fig — Top and side views of the combined palisade map before imposing C3 symmetry. (TIF) [file pbio.3002005.s010.tif]
