# Supplementary material for: A succession of two viral lattices drives vaccinia virus assembly
Source: PLoS Biol. 2023 Mar 2;21(3):e3002005. doi: 10.1371/journal.pbio.3002005 (PMC10013923; doi:10.1371/journal.pbio.3002005)
Supplement: Attachment — Submitted filename: Response to Reviewers.pdf [file pbio.3002005.s018.pdf]

Dear Richard

Thank you for your email concerning your decision on our MS "Cryo-ET of infected cells reveals that a succession of two lattices drives vaccinia virus assembly" (PBIOLOGY-D-22-02402R1) (pasted at the end of this email).

Obviously, we were a little disappointed by your decision given the very positive comments from reviewer 2, even considering the negative report of reviewer 1. I rarely rebut decisions, but feel that in this case, it is appropriate as many of the statements of reviewer 1 are not correct. Below we have responded to the reviewers' comments and concerns (also attached in PDF together with three additional response figures). I hope our arguments and responses provides you with the information you require to reconsider your decision and hopefully allow revision.

All the best

Michael

Reviewer remarks:

Reviewer #1: Hernandez-Gonzalez, et al used whole-cell cryo-electron tomography to investigate the assembly of the vaccinia virus, a model poxvirus. Poxviruses form large virions with a unique assembly mechanism involving the formation of spherical immature virions assembled from an open membrane supported by outer scaffold protein D13. The authors confirm that the D13 layer is lost during the formation of mature brick-shaped virions as shown previously. They show that D13 forms a hexagonal lattice as previously determined by various electron microscopy techniques and provide a subtomogram average consistent with the recently determined D13 scaffold atomic structure obtained from in vitro assembled D13 layer in the absence of a membrane. The transition from spherical immature to brick-shape mature virion remains enigmatic and therefore cryo-electron tomography is a suitable tool to study this process further. Authors show that the membrane of mature virion gets corrugated which is consistent with previous electron microscopy studies. Authors report interesting wire-like structures at the surface of naked cores, which were not previously reported. Overall, the manuscript is well written, mostly confirms results from previous studies and provides subtomogram averages of D13 and palisade layers at low resolution.

We are proposing membrane corrugation is the mechanism by which the viral membrane adapts to the palisade lattice that forms inside immature virions. This constitutes, together with other aspects of our model, a new idea that has not been previously suggested in the literature, in contrast to the reviewer's suggestion that this is consistent with previous EM studies (see our response to major concern 6 for a more detailed explanation).

Major concerns:

1) While it is important to apply in situ cryo-electron tomography to study vaccinia virus assembly inside the cells, authors chose to use whole-cell cryo-electron tomography, which is typically applied to study cell peripheries such as cortical actin, virus early entry events, etc. However, vaccinia virus assembly occurs in the perinuclear region, where cell thickness is higher than 500 nm. Since the transmission electron microscopy signal-to-noise ratio dramatically decreases with sample thickness, the attainable resolution and information are highly reduced. In addition, authors applied a high defocus of -8µm which due to the fringing effect skews measurements of gaps between layers reported in the work and is affecting subtomogram averaging even if CTF correction was performed. As an example, membrane bilayers of the virions or organelles are not discernable in most figures which indicates that the regions of the cells selected for the acquisition of tilt series are too

thick for high-quality cryo-electron tomography. Overall cryo-focused ion beam milling is a much more suitable sample preparation method to study the assembly of viruses in the nuclear periphery by cryo-electron tomography. Cryo-lamellae with a thickness of around 200 nm would allow using lower defocus as well as obtaining a much higher signal-to-noise ratio. Hence, authors should include cryo-electron tomography using lower defocus on cryo-focused ion beam-milled samples to support their data. As it currently stands, this work does not fully benefit from the potential of state-of-the-art in situ cryo-electron tomography.

Firstly, we fully agree with the reviewer about the fact that the majority of vaccinia virus assembly, in initial stages of infection, occurs in a perinuclear region. However, viral factories extend further in the cytoplasm as infection progresses. In our work, we took advantage of this fact to image vaccinia assembly in the periphery of cells, which is thin enough for whole-cell cryo-ET. In our MS, supplementary figure 1A, shows the specific region of the periphery of the cell imaged to produce the tomogram shown in Figure 1B, as stated in the Figure legend. This is also clearly stated in Methods (lines 389-390: *Grids were first screened on a Talos Arctica TEM (Thermo Fisher) to select edges of cells with an abundance of viral particles that were thin enough for cryo-ET*). Consequently, we did not image vaccinia assembly through thick cellular material in perinuclear regions which would hinder the resolution obtained, as suggested by the reviewer. In addition, some of the morphological forms of the virus we studied would never be found inside a cell, namely Extracellular-enveloped virions (EEV) and Cell-associated enveloped virions (CEV) due to the nature of their assembly pathway. In these cases, all virions were located and imaged outside the cell, directly embedded in the extracellular vitreous ice. In summary, in our tomograms the architecture of the membrane and palisade is the same in all mature species (IMV, IEV, CEV, EEV) regardless of whether they are inside and outside the cell. Given this, we are confident that the fact that virions are intracellular is not degrading or hindering our interpretation of the structures and architectures we describe for these virions.

We are now including the measured sample thickness of the tomogram used to produce figure 1B, showing that its ice thickness is 260-272 nm (see response figure 1 in PDF). Typically, researchers aim for below 300 nm for cryo-ET at the cell periphery. We would be happy to provide the measured ice thickness of all tomograms displayed, to show their thicknesses are around or below 300 nm.

Secondly, while collecting our data we used a magnification that results in a pixel size of 4.31 Å. We chose these magnification and defocus conditions in order to have the widest field of view with enough detail of the viral structures. When conducting cryo-ET of whole cells infected with a virus, a considerable larger field of view greatly helped us finding all stages in the cycle of poxvirus infection, something that has never been achieved before using this technique.

Thirdly, we agree that very thin cryo-FIB would help to make thin sections at the perinuclear region of the cell. However, as also explained above we were able to obtain all stages of the assembly and egress pathway of vaccinia virus focusing on the cell periphery. We would also like to remind the reviewer that vaccinia immature virions have a diameter of around 350 nm. Therefore, generating such 200-nm lamellae would not include the whole viral particle, but a physically trimmed version of it.

2) Authors use subtomogram averaging to shed light on the organization of the palisade layer of the core inside matured virions. They mention that the palisade layer is pseudohexagonal with trimeric pillars. However, the trimeric spike structure likely arises from the applied C3 symmetry during subtomogram averaging. The authors do not provide any information on how the symmetry was determined and it is not clear why was C3 symmetry chosen. Based on figure 6B the individual pillars do not appear to be trimeric. Please provide a symmetry scan result which would justify the usage of the C3 symmetry. In addition, even if the palisade pillar is trimeric the C3 symmetry should

be applied only on individual pillars and not on an array of pillars within the palisade unless the palisade layer shows a clear C3 symmetry. Using a smaller subtomogram box size or a mask that contains only a single pillar would allow obtaining more structural details of the pillar and to unambiguously determine its symmetry. In addition, authors should provide maps calculated with C1 symmetry for the palisade layers.

We thank reviewer 1 for this comment. In the Methods section of our paper we explained C3 symmetry was not applied during refinement and generation of maps of the different virion types, and C3 symmetry was only applied because the C3 symmetry of the C1 maps were evident. Nevertheless, we understand the concern of reviewer 1 and we now provide the intermediate processing data showing that C3 symmetry is apparent before imposing it. Sections of the original C1 map that combines all virion types will be shown in a supplementary figure (see response figure 2 in PDF).

3) Virion in Figure 6 shows fibrous material on the surface of the naked core. How often was this observed?

We observed this in every case we found a naked core. We thank reviewer 1 and will include this information in the paper.

4) The authors report a new outer layer on mature virions shown in Figure 5. However, this layer could represent the outer leaflet of the virion membrane bilayer. If there is a membrane below the outer layer, why is the bilayer not visible? Overall, the data quality is not sufficient to draw any conclusions about the existence of the outer layer. Again, this study would strongly benefit from including data from cryo-lamellae to provide tomograms collected on thin samples.

The presence of an outer layer covering the viral membrane that has been described as rodlet like structures/surface tubule elements that gives the virion a mulberry like appearance especially in scanning EM has been described in numerous studies. It is therefore expected that IMV will have an outer layer that is not a membrane, which we have indicated. In addition, the outer layer we describe is not present in immature virions, and only appears after IMV maturation.

Moreover, we have now provided examples in which we can see both leaflets of a viral membrane, namely the outer membrane of CEVs/EEVs (response figure 3 in PDF). The response figure 3 shows our images are close-to-focus enough to see both leaflets of smooth membranes. The protein rich IMV membranes are also corrugated in many directions, which would impede the appreciation of both phospholipid monolayers easily.

5) The D13 layer is hexagonal, however, the hexagonal lattice cannot form a sphere. Do you observe any gaps or imperfections in the D13 lattice on the immature virion which would allow for spherical curvature for example reported for the immature gag layer in retroviruses?

Detailed description of full surface lattice of an IV is not possible with our current data. Nor was a full description of the surface achieved in Hyun at al. in their in vitro system using recombinant D13 (Hyun et al 2022, PMID: 35361762). We have also not found imperfections or pentagons that could explain spherical curvature, which, have also not been found on in vitro assembled D13 spheres (Hyun et al 2022, PMID: 35361762). However, in that study pentamers were observed in flat honey-comb like patches found in vitro (see supplementary figure 1 of Hyun et al 2022). Accordingly, we mention the fact we do not observe pentamers of D13 trimers and acknowledge they have been observed before in non-spherical arrangements (Line 143 to 145: *we have not confirmed the presence of icosahedral symmetry, which would require pentameric arrangements of D13 trimers, such as those that have been identified in vitro*). We were explicit on the paper at this point.

6) The presented assembly pathway (Figure 9D) has already been proposed previously based on thin-section EM (Griffiths G et al, PMC114685; Francisco Javier Chichon et al, PMID: 19076058;). In addition, corrugated membranes and partially formed cores maturing virions are also seen in the work by Bernard Moss (PMID: 16364735) and Francisco Javier Chichon et al (PMID: 19076058), hence these manuscripts should be cited. The fact that no partially formed cores were observed makes it difficult to support the proposed assembly pathway and may reflect the rather low number of captured assembly events. This study would benefit from capturing more events.

We are proposing membrane corrugation is the mechanism by which the viral membrane adapts to the palisade lattice that forms inside immature virions. This constitutes, together with other aspects of the model we propose, a new idea that has not been mentioned or discussed before in the literature. Below, we explain why the articles the reviewer cites propose different maturation models that are not similar to the one we propose. Importantly, in no case the corrugation of the viral membrane is mentioned or given the importance we attribute to it in our model.

Griffiths et al, PMC114685: In this paper, Griffiths et al suggest a model that is still based on the idea that the core contains a membrane, and therefore IMVs are multi-membraned. We now know this is not true. Griffiths et al go even further in their model, and suggest that cores are wrapped with a cisterna coming from the endoplasmic reticulum, and that this cisterna does not fuse with itself to form two continuous membranes (later, Chlanda et al 2009 PMID: 19616767, showed D13 crescents and immature virions are formed by one single membrane that is derived from the endoplasmic reticulum after membrane ruptures), but leaves a gap that is somehow sealed by means of a protein-based system (please see in their discussion: *We have also provided evidence that the IMV cisterna does not fuse with itself to form two continuous layers of membrane around the virus but is rather folded upon itself (29). We suggested that the particle is sealed by a proteinaceous (or Velcro-like) plug that glues one cisterna upon itself***). The maturation model proposed by Griffiths et al. is radically different from the one we suggest in our paper. Ours does not involve any double-membraned cisterna, does not assume the core has a lipid membrane, and does not propose that there is a gap sealed by a Velcro-like protein, but that the single membrane that IMV have is continuous.**

Chichon et al, PMID19076058: In this article, Chichon et al propose a model drastically different from the one we show in our paper. In their maturation model, the viral membrane presents gaps or breakages through the entire assembly pathway (please see Figure 6 within). During this process, the broken pieces of membrane would also form the core of the virus. In our maturation model, the viral membrane is fully closed with no gaps or breakages, as seen by cryo-ET, and it wrinkles to adapt to the shape of the internal, non-lipid base, core, whose structure is maintained by the lattice the palisade forms.

Bernard Moss PMID16364735: In this review, Bernard Moss explores how vaccinia entry and membrane fusion occurs rather than virion assembly. However, he does discuss the number of membranes each virus type has. There is no mention of wrinkled or corrugated membranes anywhere in the text.

In writing our manuscript we were careful to read all the literature and examine images to ensure the features of our model had not been described previously. We are confident we did not miss anything and indeed during this analysis we actually found some published thin section EM images suggesting the existence of the IV invaginations we have described in figure 1 of our MS that were not actually previously mentioned. That is why we cite the reference in line 275.

7) Please report how many immature virions show a single invagination of the viral membrane in Figure 1B.

Based on our images a large proportion of IV have an invagination. In the revised paper we will provide a detailed quantification to address the reviewer's question.

8) How was the resolution of 125 nm in structured illumination microscopy determined? This method does not seem to be described in the methods section.

In the revised paper we will provide the full information and details requested by the reviewer. However, basically, we measure the point spread function (PSF) of sub diffraction beads (100nm) to confirm that the resolution of the imaging system meets the 125nm specification after deconvolution.

9) Supplementary Figure 3B has missing panels.

We did not see an issue in the paper we uploaded and in MS downloaded from the journal site after submission we see no loss of figures when the PDF is viewed with Adobe.

10) The authors used Vitrobot for the vitrification of infected cells grown on grids. Was double-side blotting applied prior to plunge-freezing?

In the revised paper we will provide the full information requested by the reviewer but basically 4 ul of PBS containing gold fiducial markers were pipetted on the sample side of the grid. Then, it was blotted using vitrobot on both sides.

Reviewer #2: This manuscript is about the structure of the intracellular immature poxvirus particle (IMV), determined by CET. The main discovery is a pseudohexagonal palisades layer that coats the viral core, and the hypothesis that the length of the core particle is determined by the D13 lattice of the immature virion, while the palisades layer controls shape and dimensions of the virion during assembly. Tomographic reconstructions from various snapshots of the virion maturation process are presented. This may be the first comprehensive description of various states during maturation (IV, IMV, IEV, CEV) by CET.

This is a progressive manuscript, which describes and boldly interprets several novel observations. The authors provide a comprehensive introduction listing relevant references.

The study of the vaccina virus maturation process of cryo-fixated samples using CET is a major advance over previous studies using traditional chemically fixated, plastic embedded and heavy-metal-stained samples, which are much more prone to inducing artifacts.

We thank the reviewer for their positive comments and appreciation of the quality and importance of our study.

Fig. 2A clearly shows the in-situ structure of the D13 lattice in the IV, including a gap separating the D13 layer and the lipid membrane underneath. Any possible connecting densities, e.g., by A17, are not resolved, as stated by the authors. This could be due to irregular incorporation of A17.

Elongated structures in the IV are interpreted as DNA inside viral factories.

Reconstruction of the IMV reveals a novel palisades layer, which is nicely shown in detail in multiple

figure panels in different maturation states. The palisade was reconstructed by subtomogram averaging, which should remove any artifacts due to missing wedge data.

I have no major concerns.

Denoising with ISONet, which "fills in" missing wedge data, was used only on final reconstructions used for segmentations.

In the revised paper we will comment on the specific use of ISONet, which was only used on final reconstructions used for segmentation. It was not used during sub tomogram averaging.

The discussion is somewhat speculative.

We think it is important to discuss the implications from our results and propose new ideas that open new avenues for future research (see below).

Minor points (suggestions to the authors):

Is there any biological relevance to the naked core particles of purified IMVs? These are introduced without further explanation besides their morphology. How do they fit into the maturation process?

We thank reviewer 2 for this comment, which we believe refers to cut corners (lines 184-185) in results. We mention this information to illustrate that the shape of the core (cut corner) is an intrinsic structural property of the palisade and not something that is imposed by the viral membrane. We will give more context to this comment in the revised version of the manuscript.

I feel less convinced about the interpretations of invaginations and related DNA entry sites. The speculation (line 279) should be removed.

We understand this is a speculation, and that is why we wrote in the way: *"It is tempting to speculate…"*. We understand the reviewer thinks it is too speculative and are willing to remove or tone down the text. However, given the frequency of invaginations we believe there is likely to be a connection with DNA insertion as its not obvious what other process would generate a single invagination.

Line 136: the resolution of the tomogram (Fig. 3B) does not seem to allow making any statements about the relative twist between the docked D13 trimers - this sentence should be removed.

Line 136: We are happy to remove this line.

Line 360 seems unnecessary and simply caters towards the current hype about MPX.

Line 360: We think the strong conservation of both the replication cycle and the proteins involved in poxviruses might have passed inadvertently to those readers not expert on this virus family. Because vaccinia is used as a model virus in the family, we think it is important to put in context the importance of the research we carry out. Therefore, we would like to maintain this line.

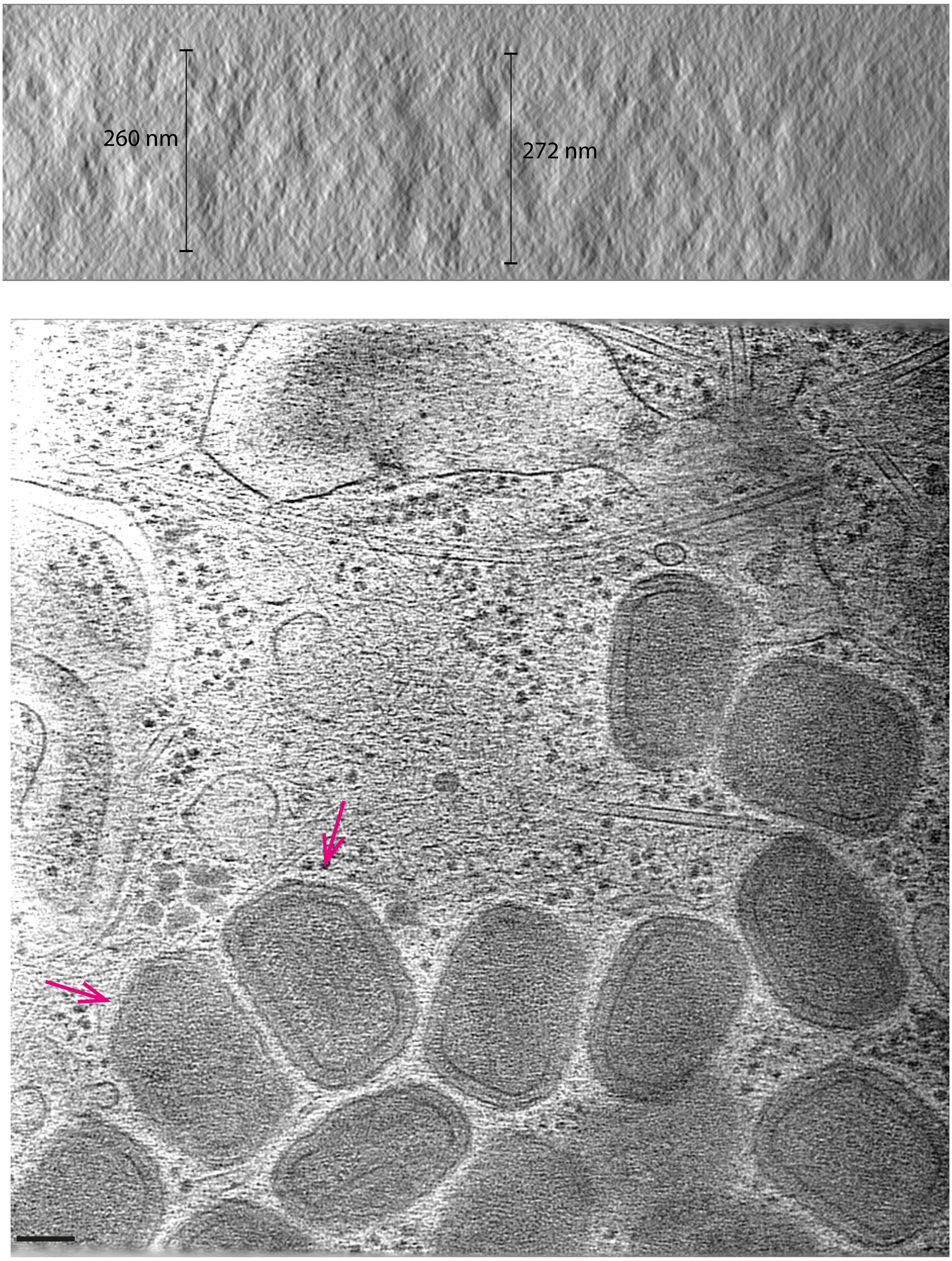

Ice thickness of an intracellular cryo-ET tomogram. Top image shows a side view of the tomogram that shows intracellular IMV particles in Figure 1. The ice thickness is around 260-270 nm. Scale bar = 100 nm.

Response Figure 1

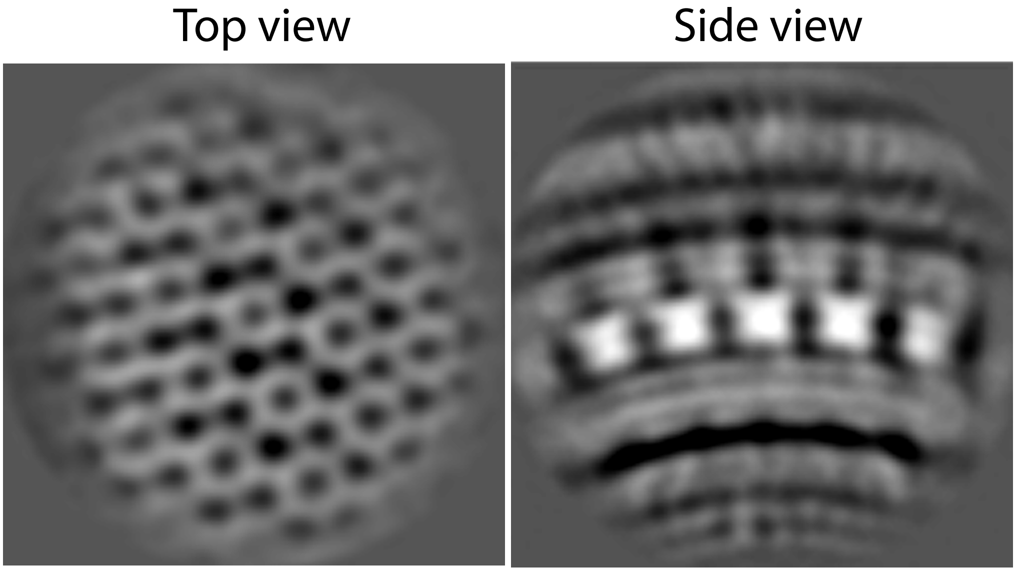

Combined palisade map, C1 symmetry. Top and side view section of the combined palisade map before imposing C3 symmetry.

Response Figure 2

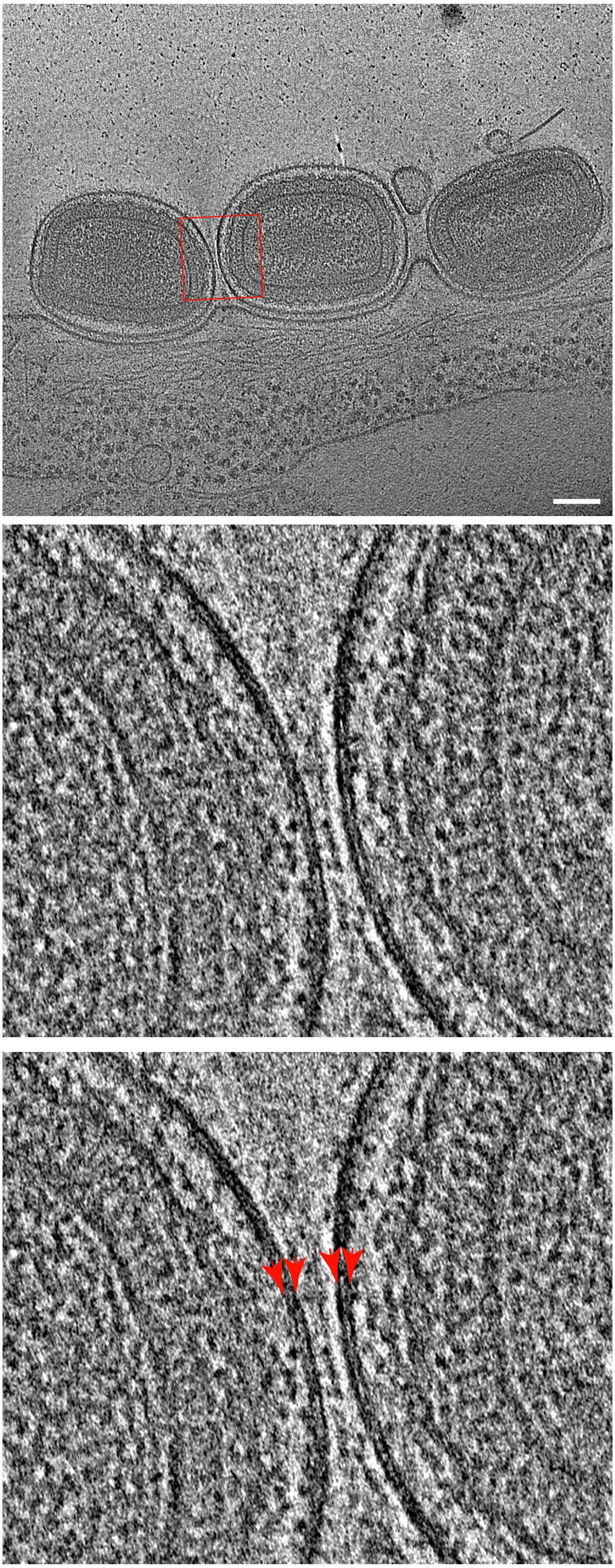

Tomogram section of CEV particles. The CEV outer membrane is seen as two parallel densities, according to the bilayer nature of biological membranes. Scale bar = 100 nm.

Response Figure 3
